# Escalating frustration - A replication attempt and extension of Yu et al. (2014)

**DOI:** 10.12688/openreseurope.17749.2

**Published:** 2025-04-10

**Authors:** Charlotte Eben, Zhang Chen, Raquel E. London, Frederick Verbruggen

**Affiliations:** 1 Department of Experimental Psychology, Ghent University, Ghent, Flanders, 9000, Belgium; 2 Department of Psychology, The University of British Columbia, Vancouver, British Columbia, V6T 1Z4, Canada; 3 Department of Biology, Ghent University, Ghent, Flanders, 9000, Belgium

**Keywords:** frustration, reward, response force, facial EMG

## Abstract

**Background:**

Failures to obtain a desired reward, such as losing money in gambling, can lead to frustration. In gambling, this frustration has been shown to take the form of faster responses after losses compared with wins and non-gambling trials. In addition, reward omission or blockage can lead to more forceful responses. Yu and colleagues (2014) showed that the proximity to a reward and the effort already expended to acquire the reward increased participants’ response force and their retrospective self-reported frustration when the reward was blocked.

**Methods:**

In this study, we attempted to replicate the findings of Yu and colleagues (2014) using the same experimental procedure. In each schedule, participants (N = 32) needed to complete an arrow direction task for varying numbers of times to win a reward but could be blocked at any stage. The response time (RT) and force of confirming the outcomes were used as indicators of ‘frustration’. In addition, to obtain a more real-time and objective measure of (negative) emotion, we measured facial electromyographic (EMG) activity over the corrugator supercilii (frowning muscle) and the zygomaticus (smiling muscle).

**Results:**

Due to technical problems, our data on response force were invalid. In line with the original study, both goal proximity and exerted effort increased participants’ self-reported motivation in the task and frustration after being blocked. An exploratory analysis showed that unexpectedly participants were slower in confirming an outcome when they were blocked closer to the reward, while exerted effort did not influence the time taken to confirm the outcome. These RT data were consistent with self-reported surprise ratings, suggesting an orienting response. In the facial EMG data, we observed no difference between wins and losses in activity over the corrugator or the zygomaticus.

**Conclusion:**

Taken together, these data suggest that reward blockage does not necessarily lead to behavioral or psychophysiological expressions of negative emotions such as frustration.

## Introduction

Humans and non-human animals generally try to achieve desired goals. Along the way, however, they may encounter obstacles and be prevented from achieving these goals: for example, in birds, a competitor may take a valuable piece of food before the individual has the opportunity to eat; or in humans, we may not get the promotion that we think we deserve. To understand goal-directed behaviour, it is important to understand how individuals respond to such failures to achieve desired goals.

Both examples above may lead to ’frustration’: When an expected reward is omitted, individuals sometimes respond with more vigor (e.g., speed, force), which is called the ’frustration’ effect. For example, Amsel ( 1958, 1992); observed that rats that were typically rewarded in a first runway of a maze ran through the second runway faster if the reward was not given in the first runway. Furthermore, he observed more aggressive behavior, such as urinating or biting the experimenter, after reward omission. Similar effects have been observed in other species, such as fish ( Vindas *et al*., 2012), mice ( de Almeida & Miczek, 2002), laying hens ( Zimmerman & Koene, 1998) and pigeons ( Zentall, 2011). Variations of this effect have been observed in humans as well. For example, Verbruggen *et al*. (2017) showed that participants tended to initiate a new trial more quickly after losses in gambling tasks compared to wins and non-gambles, which was interpreted as a manifestation of the ’frustration’ effect (for direct and conceptual replications of this finding, see Eben *et al*., 2020). Speeding up after losses has recently also been observed in large behavioral tracking data from a real online gambling product ( Chen *et al*., 2022).

Many goal pursuit activities require individuals to carry out multiple successive actions to obtain a reward. Accordingly, individuals may fail at any stage of the goal pursuit process, which in turn may influence the magnitude of the ’frustration’ effect triggered by reward omission or blockage. As individuals make progress in their goal pursuit, their motivation to obtain the goal generally increases (i.e., the goal gradient hypothesis; Hull, 1932). Recent work has shown that people generally exert more cognitive effort when they are provided with information on their progress in a task ( Devine & Otto, 2022; Katzir *et al.*, 2020), and cognitive effort exertion increases near the end of a task (i.e., near goal completion, Devine *et al.*, 2024; Emanuel *et al.*, 2022). Outside the laboratory context, the influence of perceived goal progress on motivation has also been documented. For instance, customers bought coffee more frequently the closer they were to getting a free coffee by collecting enough stamps in a reward program ( Kivetz *et al.*, 2006). In addition to influencing people’s motivation, goal progress can also influence the magnitude of the ‘frustration’ effect when they are blocked from the reward ( Amsel, 1958). For instance, Haner and Brown (1955) found that children responded with more force when a failure occurred close to success, compared to when they were still far from achieving their goal.

The ubiquitous influence of goal progress on motivated behavior requires a better understanding of how such influence arises. Here we specifically focus on how goal progress may influence the magnitude of the ’frustration’ effect when individuals are blocked from a reward. Two closely interrelated factors seem crucial in the effects of goal progress, namely expended effort ( Pompilio *et al*., 2006) and goal proximity ( Shidara & Richmond, 2002). As individuals make progress in their goal pursuit, the effort already invested increases (i.e., expended effort), while they also get closer to the end reward (i.e., goal proximity). In many situations, these two factors are inherently confounded, but the psychological mechanisms underlying the effects of these two factors may differ. For instance, expended effort is retrospective in nature as it concerns the effort already invested, while goal proximity is prospective as it orients towards an upcoming reward ( Yu *et al*., 2014). Disentangling the effects of expended effort and goal proximity will therefore be informative in understanding successive goal-directed behaviors, and how individuals react when they fail.

Yu *et al*. (2014) developed an experimental task to disentangle these two typically confounded factors (see Method for more details). The task consisted of multiple ’schedules’. In each schedule participants could win a monetary reward, indicated by an image of a coin divided into four parts. To get the monetary reward, they needed to obtain all four parts of the coin, thus each schedule constituted one goal-pursuit attempt with multiple successive actions. In each schedule, either no part or some parts of the coin were already given to participants for ’free’, so the number of ’trials’ varied from one (i.e., only one quarter of the coin was missing) to four (i.e., the whole coin was missing). To obtain a missing part of a coin in a trial, participants had to do a task in which they indicated the left versus right direction of arrows via button presses. At any stage however, they could get blocked. That meant that even when the response was correct, the participants were told that they were too slow and that they would not get the reward. By manipulating the number of trials in a schedule and at which stage participants were blocked, Yu *et al*. (2014) were able to disentangle expended effort and goal proximity. For example, when participants were blocked at the very first trial out of four trials, both goal proximity and expended effort were low; when they were blocked in the last trial out of four trials, both goal proximity and expended effort were high (i.e., the typical situation with the two factors confounded). However, when participants were blocked in the first trial in a one-trial schedule (i.e., three quarters of a coin were already available, the schedule consisted of only one trial), goal proximity was high (as participants were close to obtaining the reward) but expended effort was low (as they only did one trial). Using this procedure, Yu *et al.* (2014) showed that goal proximity and expended effort independently influenced participants’ self-reported motivation and the response times (RT) in the arrow task, as well as their self-reported frustration and the force with which they confirmed an outcome when they were blocked from obtaining a reward. More concretely, participants reported being more motivated and responded faster in the arrow task the closer they were to their goal and the more effort they had expended to reach the goal. When they were blocked from obtaining a reward, both the self-reported frustration (collected retrospectively at the end of the experiment) and the response force with which people confirmed the outcome increased as goal proximity and expended effort increased. Taken together, this study shows that goal proximity and expended effort, two frequently confounded factors, independently contribute to the motivational and emotional influences of goal progress, affecting both successive goal-pursuit behaviors and how people react to outcomes (e.g., reward blockage) of their reward pursuit attempts.

The task by Yu *et al*. (2014) successfully disentangled the motivational and emotional influences of expended effort and goal proximity during reward blockage, two important yet frequently confounded factors. As this could be a very valuable tool for studying reward blockage and frustration, we aimed to replicate and extend the original study by Yu *et al*. (2014). Yu *et al*. (2014) measured experienced frustration only retrospectively at the end of the experiment with self-reports. To further explore the emotional influences of reward blockage, we extended Yu *et al*. (2014) by measuring facial muscle activities throughout the experiment with facial electromyography (fEMG). More specifically, we measured the activities of the corrugator supercilii (the muscle that is involved in frowning) and zygomaticus major (the muscle involved in smiling). When being presented with negative stimuli, normally the corrugator supercilii is active and when being presented with positive stimuli, usually the zygomaticus major is active (e.g., Mauss & Robinson, 2009). The activities of these two facial muscles thus provide a measure of the positive versus negative valence of experienced emotions *while* participants engage in the task, instead of retrospectively. This online measurement of valence has been used in previous studies to investigate the evaluation of gambling outcomes ( Wu & Clark, 2015), which found heightened activity of the corrugator supercilii after losses. Furthermore, fEMG has been used to investigate outcomes in cognitive tasks, and found that errors (which are presumably negative outcomes) similarly increased the activity over the corrugator supercilii ( Elkins-Brown *et al*., 2016; Lindström *et al*., 2013).

Taken together, the current study had two aims. The first aim was to replicate the original findings by Yu *et al.* (2014), that expended effort and goal proximity would lead to shorter response times and higher self-reported motivation in the task, and more response force and higher self-reported frustration when participants were blocked from a reward. For this, we used the same task as Yu *et al*. (2014) and measured RT and response force with a custom response box, and self-reported frustration, surprise and motivation at the end of the experiment. Furthermore, we measured activity over the corrugator supercilii and the zygomaticus major in an exploratory manner. Note that some unexpected issues with the response box made our measurement of response force invalid. We reported, however, the results of the force measurement for consistency and transparency in the online supplementary materials ( https://osf.io/6v9ak) and discussed the issue of the response box in the discussion.

## Methods

### Transparency and openness

All raw and processed data, code, and materials can be found on OSF ( Eben *et al*., 2024, https://osf.io/rmh2d/). We did not preregister the analyses as we did not have experience with measurements of force or facial EMG. We did however decide on a data analysis approach before looking at the data. In this manuscript we clearly report which analyses were planned and which analyses were added as exploratory analyses. We report how we determined our sample size, all data exclusions, all manipulations, and all measures in the study. The research was approved by the Ethics committee of the Faculty of Psychology and Educational Sciences of Ghent University (ethical approval number 2022-135; approved 17/01/2023). Thus, the study adhered to the Declaration of Helsinki. Participants provided written informed consent before starting the experiment.

### Sample size

In the original analysis, Yu and colleagues examined the effects of proximity and expended effort by combining the p values from multiple repeated-measures ANOVAs using the Stouffer’s method. As far as we know, no analytical method exists to compute the statistical power for the Stouffer’s combined p values. We therefore decided to go beyond the sample sizes used in Yu *et al.* (between 20 and 27), but still within our resource constraints. We were additionally interested in potential differences in the response force after wins and losses, for which Yu *et al.* did not report the results. We therefore assumed a medium effect size (Cohen’s d = 0.5), for which 32 participants would provide approximately 80% power to detect in a within-subject design. Our response force data, however, was compromised and we never followed up on that specific effect. The eventual sample size of 32 was therefore chosen given all these considerations, to (1) exceed the original sample sizes, (2) be within our resource constraints, and (3) provide sufficient power to detect potential response force differences between wins and losses.

### Participants

In total 32 participants (recruited via the local Sona system from Ghent University) completed the experiment between March and July 2023 and remained in the final analysis (23 females, 1 non-binary; 3 left-handed; age *M* = 24.53 years, *SD* = 3.38 years, *range* = 18–33). Participants were paid at a rate of 10€/hour plus a bonus (see below) of 10€. As this experiment took approximately 90 minutes (30 minutes of preparation plus 60 minutes on the task), they received 25€ (1.5*10€ +10€) in total.

### Apparatus and stimuli of experimental task

The experiment was programmed in PsychoPy (version 2022.2.4; Peirce, 2007). A custom response box was used to register response latency and force with a sampling frequency of 200Hz. The box had two response keys (i.e., mechanical key switches in keyboards), which registered responses and RTs when pressed. Flexiforce sensors on top of the keys were attached to measure the response force. The Flexiforce measuring range had been fixed at 2500g, with 2.45g measuring steps (via Arduino ADC). Even though we tested the set-up before the experiment, post-hoc inspection of the force data occasionally revealed force value of 0 for some of the participants, even though a response (and the corresponding response latency) was registered by the box. As responses were registered using keys similar to those of a keyboard and force was measured with sensors on these keys, it is possible that participants pressed the button without fully placing their index fingers on the sensors, despite being instructed to do so. Therefore, we cannot exclude that all our force data is underestimated and therefore invalid. For consistency and transparency reasons, we report the force data in the online supplementary materials ( https://osf.io/6v9ak) but we will not discuss their theoretical implications.

### Procedure

**Figure 1.**
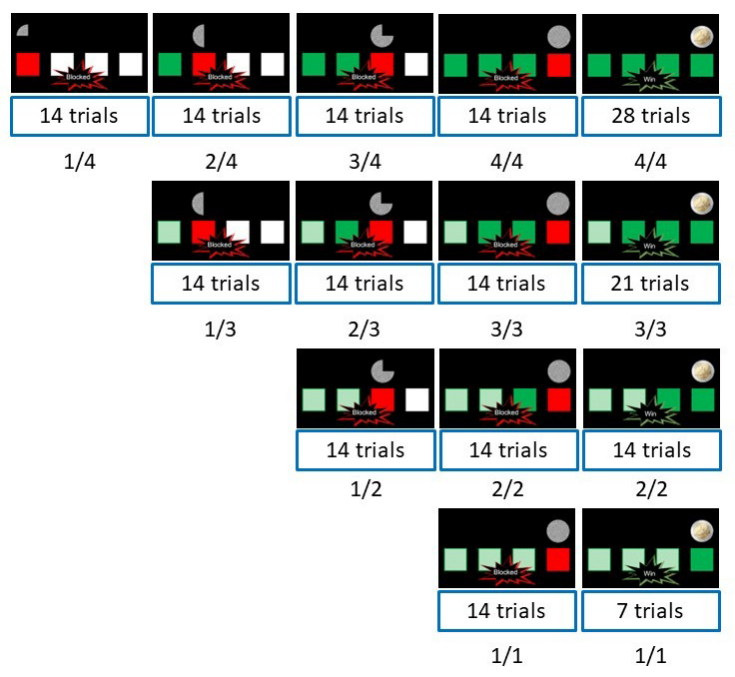
The different schedules used in the experiment. The outcome screens of the different schedules used in the experiment. Note that the screenshots here do not represent the progression across trials within a schedule, but rather only the outcome screen at the end of a schedule. Blocked 1/4 means that the schedule consists of 4 trials, and participants were blocked at trial 1. The numbers within the boxes indicate the number of each specific schedule included in the experiment.

We used the same multi-trial reward schedule task developed by Yu *et al*. (2014). At the beginning of each schedule, participants were presented with a cue for 2000 ms (see video on Open Science Framework: https://osf.io/z28b4). The cue consisted of four boxes that were filled with either green stripes (light green boxes in Figure 1) or white color. Depending on how many trials needed to be completed, either no part or some parts of the coin were visible (e.g., if participants had to complete four trials, no part of the coin was visible; if they had to complete three trials, a quarter of the coin was already visible etc.). The number of boxes with green stripes indicated how many parts of a coin were given to participants ’for free’. The number of white boxes indicated the amount of trials to complete in order to obtain the reward (varying from 1 to 4 trials across schedules). After the cue screen, a blank screen was shown for 750 ms, followed by the arrow task. In the arrow task, participants were presented with three white arrows either all pointing to the left or to the right, and needed to indicate the direction by pressing the left or right button. The arrows were presented for 1 second. Participants were instructed to respond as fast and accurately as possible. We told participants (as in the original study) that on each trial, the computer randomly determined a RT criterion that they would have to meet, otherwise they would be blocked and not receive the reward. In fact, whether participants would be blocked or not on a particular trial was pre-determined (see Figure 1 for the predetermined schedules). Furthermore, if participants made a mistake or were too slow (i.e., RT > 500 ms), they would be blocked regardless of the pre-determined outcome of a trial.

If participants were not blocked on a trial, one white box became green and an additional quarter of the 2 euro coin image became visible. The cue was again shown for 2 seconds, and participants received a new arrow task after a blank screen of 750 ms. As long as participants were not blocked, this sequence of events was repeated until the end of a schedule. In that case, all white boxes were filled green, and the whole 2 euro coin was visible. A text message "Win" was superimposed on the cue, to indicate the outcome of the current schedule. If participants were blocked at any stage, the square for the current trial became red, and the coin image was replaced by an image of white noise (see Figure 1). A text message "Blocked" was superimposed on the cue and the current schedule ended immediately (that is, participants did not need to do the remaining trials in a blocked schedule). In both cases (win and blocked), participants then needed to confirm the current outcome. In the confirm task, they were either presented with ’win? blocked?’ with the left button press to confirm a win trial and the right button press to confirm a blocked trial, or with ’blocked? win?’ with a left button press to confirm a blocked trial and a right button press to confirm a win trial. As in the original study, participants were instructed to take their time to give the confirm response to avoid having response speed confounding the response force. The confirmed screen was presented for four seconds, regardless of the participants’ confirmation RT.

The experiment started with a practice block of 5 schedules, followed by seven experimental blocks of 30 schedules (210 experimental schedules in total). We used the same hidden schedule structure as in Yu *et al*. (2014) (see Figure 1). Participants were told that at the end of the experiment, the program would randomly pick 10 schedules. For each win outcome, they would receive 2 euros. For each blocked outcome, they would receive no extra money. We told them that the results of these 10 selected schedules would be added up, to determine their final bonus (with 10 euros as the maximum) when in fact, all participants received the 10€ bonus. During the self-paced break after each block, participants were asked to tell the experimenter when they wanted to continue, as this could only be done with the computer keyboard.

At the end of the experiment, participants indicated how frustrated they felt at different schedule stages using a 10-point Likert scale (1 = not at all, 10 = very intensely). For this, we presented the different schedules as images and asked participants to click on the Likert scale to indicate their frustration level. They also indicated how surprised they felt after being blocked at each schedule stage and how motivated they were to complete a schedule after seeing the different cues. They then filled in a handedness questionnaire (which can be found in the supporting docs folder on Open Science Framework). Participants were debriefed, and all received 25 euro as compensation.

### Electrophysiological recording and data reduction

According to common guidelines and practices ( Fridlund & Cacioppo, 1986; Korb *et al.*, 2017), we attached shielded EMG electrodes over the right zygomaticus major and corrugator supercilii areas and placed a ground electrode at the top of the central forehead. Using the Biopac EMG100C amplifier with the MP150 module and the Acqknowledge software ( www.biopac.com), we acquired continuous EMG at 1000 Hz sampling rate, with a 100 Hz high-pass and a 500 Hz low-pass.

The data reduction and preprocessing pipeline was based on Korb *et al.* (2017), van Boxtel (2010) and Whelan (2003), and adjusted for our design. We applied a 20-200 Hz band pass filter and full-wave rectified the data by flipping all negative values of the signal to positive values. To exclude high-frequency noise, we then applied a 40 Hz low-pass filter. We epoched the data time-locked to the outcome presentation (win or block) from -750 ms to 2000 ms. Before cleaning the data, we compared the behavioral and EMG data to identify extra "ghost" triggers (triggers that Biopac picked up but were never sent) and deleted these. Then, we excluded error trials and trials where the baseline was more than 2 standard deviations from the participant’s baseline mean. The signal was normalized by expressing it as a percentage of the baseline mean per trial. This was done to ensure comparability between individuals and muscles, as well as to control for changes in muscle tone over time. Finally, we excluded the epochs with responses two standard deviations higher than the participant’s condition mean.

### Planned analyses

***Confirm RT measures and force data.*** All behavioral data processing and analyses were completed with R ( R Core Team, 2013, version 4.2.0) using the packages ez ( Lawrence, 2016, version 4.4-0), poolr ( Cinar & Viechtbauer, 2022, version 1.1-1), lmer ( Kuznetsova *et al*., 2020, version 3.1-3), report ( Makowski *et al*., 2023, version 0.5.7), extraDistr ( Wolodzko, 2023, version 1.10.0), loo ( Vehtari *et al*., 2023, version 2.6.0), bridgesampling ( Gronau *et al*., 2020, version 1.1-2), brms ( Bürkner, 2017; Bürkner, 2018; Bürkner, 2021, version 2.20.4), bayesplot ( Gabry & Mahr, 2024; Gabry *et al*., 2019, version 1.11.0), bayestestR ( Makowski *et al*., 2019, version 0.13.1), tidaybayes ( Kay, 2023, version 3.0.6) tidyverse ( Wickham, 2021, version 1.3.2) and PerformanceAnalytics ( Peterson & Carl, 2020, version 2.0.4).

We excluded and replaced two participants where we had problems with data recording, one participant where a memory error in PsychoPy prevented us from finishing the testing, two participants where the electrodes fell off during testing, one participant who seemed to have not understood the task and told us they adopted a guessing strategy, and four participants with fewer than 7 observations in at least one cell after excluding error trials.

For the analyses, we excluded all practice trials, trials in which participants made an error or the RT was above 500 ms, and schedules in which the confirm response (win vs. block) was incorrect. We also excluded trials with an RT of 0 ms as this was an impossible value. The analyses focused on the effect of getting blocked on RT; thus, we only analyzed trials in which participants got blocked. In line with Yu *et al*. (2014), we tested a proximity effect and an effort effect. For the proximity effect, we conducted three univariate ANOVAs: one ANOVA compared schedules on which participants got blocked in the first trial (i.e., 1/1, 1/2, 1/3, 1/4). For these schedules, the effort was held constant at a level of 1 but the proximity could vary between 1 and 4. The second ANOVA compared all schedules on which the participants got blocked in the second trial (i.e., 2/2, 2/3, 2/4). Here the effort level was constant at 2 but the proximity could vary between 1 and 3. The last ANOVA for the proximity effect compared all schedules on which participants got blocked on the third trial (i.e., 3/3, 3/4). Here, the effort level was constant at 3 and the proximity could vary between 1 and 2. To calculate the statistical significance level for the overall proximity effect, Stouffer’s combined p ( Whitlock, 2005) was computed as it had been done in the original study. This procedure was applied to all dependent variables, including RT to the arrows, confirm RT, frustration ratings, surprise ratings and motivation ratings. The analyses on the confirm response force can be found in the online supplementary materials.

For the effort effect, we also conducted three ANOVAs per dependent variable: keeping the proximity constant at a level of four, we compared all trials on which the participants got blocked on the last trial of a schedule (i.e., 4/4, 3/3, 2/2, 1/1). Here the effort level varied between 4 and 1. The second ANOVA compared all schedules on which the participants got blocked on the penultimate trial (i.e., 3/4, 2/3, 1/2). Here the proximity level was constant at 3 but the effort level varied between 3 and 1. Lastly, we compared all schedules on which the participants got blocked on the antepenultimate trial (2/4, 1/3). Here, the proximity level was 2 and the effort level varied between 2 and 1. Again, we used Stouffer’s p to combine all three p-values into one index for the effort effect for each dependent variable.

Furthermore, as in the original study we calculated Pearson’s correlations between the mean RT in the arrow task, the mean RT of confirming an outcome (after being blocked), and the self-reported motivation, frustration, and surprise ratings in different schedules. For this analysis, we computed the mean score of each variable per schedule and collapsed the data across all participants. In other words, schedules rather than participants were the unit of analysis. All correlations including the response force can be found in the online supplementary materials.

We report Stouffer’s combined p-values and p-values for statistical inferences. The alpha level was .05.

***facial EMG data.*** The facial EMG analyses were conducted with python ( Python Software Foundation, 2000, version 3.9.18) using the libraries numpy ( Harris *et al*., 2020, version 1.24.3), matplotlib.pyplot ( Hunter, 2007), bioread ( Vack, 2023, version 3.0.1), mne ( Gramfort *et al*., 2013, version 1.5.1), scipy ( Virtanen *et al*., 2020, version 1.11.1), and pandas ( pandas development team, 2020, version 2.0.3). The individual event related potentials (ERP’s) were created by averaging the win trials and all conditions of the blocked trials for each muscle and individual. This resulted in 4 ERP’s per individual (2 types of outcomes, and two muscles). Note that we did not distinguish among the different win and blocked schedules, to maximize the number of trials per condition. As depicted in Figure 1, we did not have enough trials per individual schedule for fEMG analyses. The Grand Average ERP’s were created by averaging over individual ERP’s, resulting in 4 ERP’s total. To test for significance, for both muscles we repeated a 2-sided t-test at each time-point comparing win to block conditions using an alpha of 0.05. We controlled for multiple comparisons with a cluster-based permutation test ( Maris & Oostenveld, 2007). All data-points with a t-value exceeding the threshold (corresponding to a probability < 0.05) were clustered based on adjacency in the temporal domain. For each cluster the sum of t-values was then calculated and the maximum of these cluster-level statistics was taken. To create a reference distribution against which to test the value of this cluster-level statistic, 5000 permutations of the data were conducted. Each iteration yielded a maximum cluster level statistic and over iterations a null distribution of maximum cluster level values was constructed. The p-value of the effect was then estimated as the proportion of elements in the null distribution exceeding the observed maximum cluster-level test statistic.

## Results

### Original analyses - Stouffer’s p

**Figure 2.**
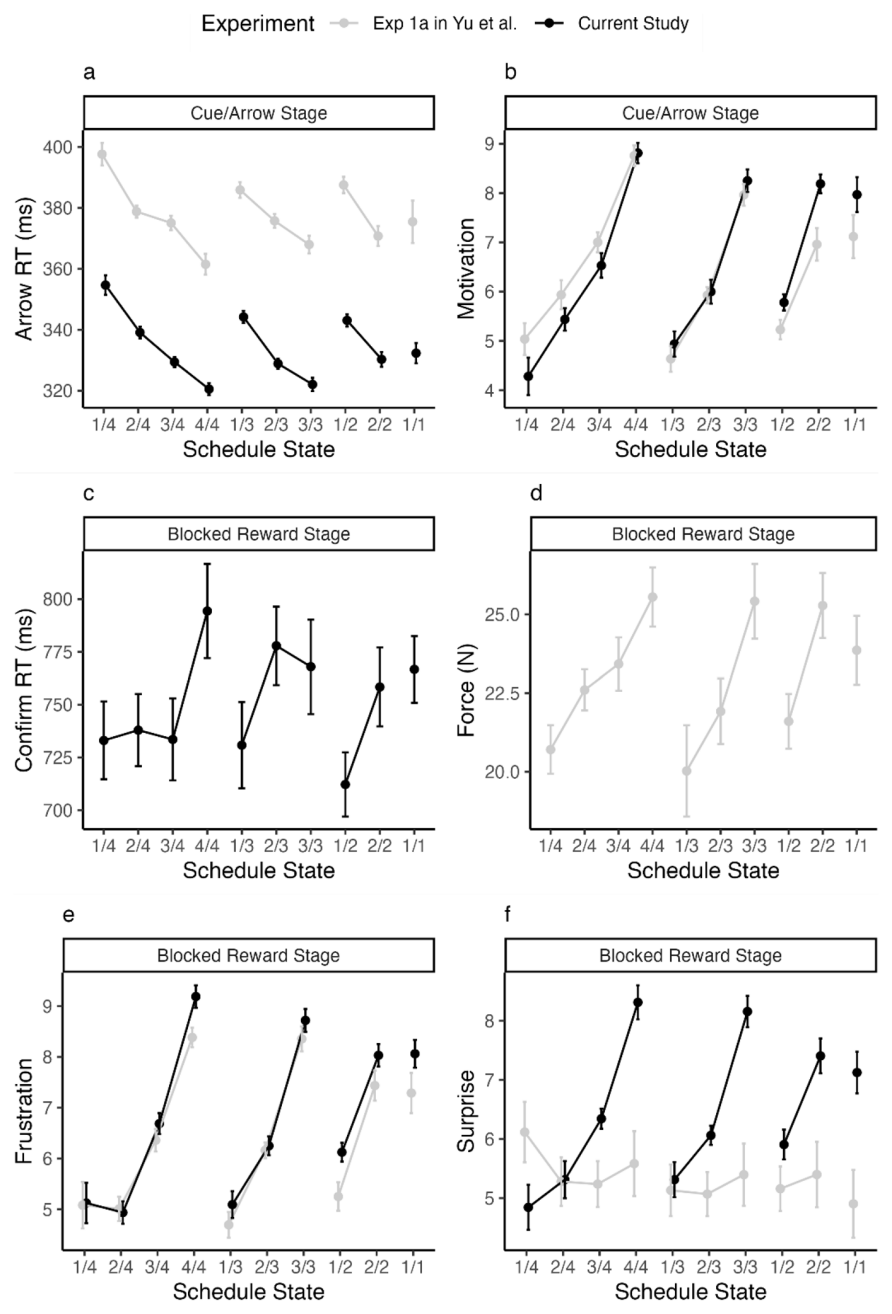
Behavioral and self-report data. Behavioral and self-report data. ( **a**) RTs of correct responses to arrows, ( **b**) self-reported motivation for each cue during the arrow task, ( **c**) confirm RTs and ( **d**) response force after being blocked, ( **e**) self-reported frustration and ( **f**) self-reported surprise at being blocked. Data for plotting the results of Yu *et al.* were extracted from their Figure 3 using plotdigitizer.com. Note that for ( **c**) confirmRTs, Yu *et al.* did not plot the results. For ( **d**) response force, our force data was invalid and thus not shown. Error bars indicate within subject standard errors.

The descriptive results can be found in Figure 2. In line with the original study, the RT to the arrows showed a significant decrease as a function of goal proximity (Stouffer’s *p* < 0.01) and increased effort (Stouffer’s combined *p* < 0.01). Similarly, in line with the original study, the confirm RT did not show any significant increase as a function of goal proximity (see Figure 2; Stouffer’s combined *p* = 0.13) or increased effort (Stouffer’s combined *p* = 0.28). Similarly, subjective emotional ratings completed after the experiment showed that participants’ frustration increased with increased goal proximity (Stouffer’s combined *p* < 0.01) and increased effort (Stouffer’s combined *p* < 0.01). Motivation ratings also increased with goal proximity (Stouffer’s combined *p* < 0.01) and increased effort (Stouffer’s combined *p* < 0.01). In contrast to the original study, the surprise ratings also increased with goal proximity (Stouffer’s combined *p* < 0.01) but not as a function of increased effort (Stouffer’s combined *p* = 0.99). All results from the single ANOVAs can be found in Table 1.

**Table 1.** The *F* values and *p* values from the ANOVAs on the effects of proximity and effort across 4, 3, and 2 trials. Stouffer’s combined *p* values are reported as an index of the significance of combined effects of proximity or expended effort across the three ANOVAs.

Conditions	arrow RT	Motivation	confirm RT	Frustration	Surprise
**Proximity effect** 1/4, 1/3, 1/2, 1/1	14.32 ( *p* < 0.001)	27.62 ( *p* < 0.001)	1.78 ( *p* = 0.156)	24.42 ( *p* < 0.001)	10.15 ( *p* < 0.001)
2/4, 2/3, 2/2	9.29 (p < 0.001)	37.31 ( *p* < 0.001)	1.12 ( *p* = 0.332)	49.99 ( *p* < 0.001)	14.69 ( *p* < 0.001)
3/4, 3/3	1.04 (p = 0.315)	31.02 ( *p* < 0.001)	1.04 ( *p* = 0.315)	53.18 ( *p* < 0.001)	57.29 ( *p* < 0.001)
combined effect	*p < 0.001*	*p* < 0.001	*p* = 0.133	*p* < 0.001	*p* < 0.001
**Effort effect** 4/4, 3/3, 2/2, 1/1	6.35 (p < 0.001)	3.64 ( *p* = 0.015)	0.68 ( *p* = 0.564)	9.46 ( *p* < 0.001)	5.39 ( *p* < 0.001)
3/4, 2/3, 1/2	19.07 (p < 0.001)	4.35 ( *p* = 0.017)	3.88 ( *p* = 0.026)	2.63 ( *p* = 0.080)	1.33 ( *p* = 0.272)
2/4, 1/3	3.47 (p = 0.072)	7.29 ( *p* = 0.011)	0.07 ( *p* = 0.792)	0.42 ( *p* = 0.523)	< 0.01 ( *p >* 0.999)
combined effect	*p < 0.001*	*p* < 0.001	*p* = 0.287	*p* < 0.001	*p* = 0.993

### Original analyses - correlational analyses

**Figure 3.**
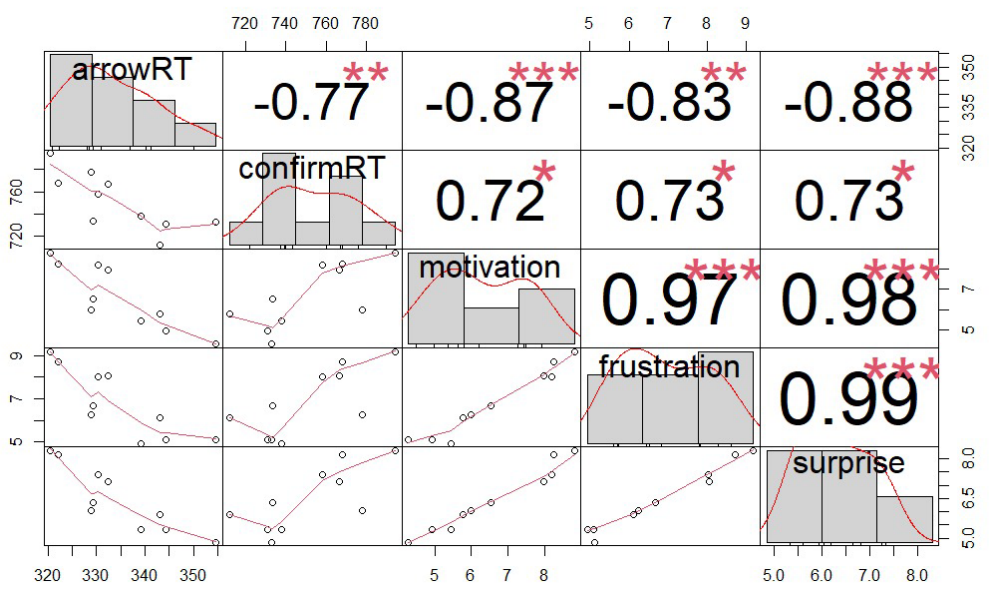
Correlations between the mean RT in the arrow task, the mean RT of confirming a blocked outcome, and self-reported motivation, frustration and surprise in different schedules. The numbers above the diagonal show the correlation coefficients. ***p < .001, **p < .01, *p < .05.

For completeness, we present the whole correlation matrix in Figure 3 but only describe the correlations that were reported in the original study here. In line with Yu *et al*. (2014), we found a significant correlation between mean RT on the arrow task in the 10 schedule stages and mean self-reported motivation (r = -0.87, *p* < .001). Participants reported a higher motivation to do the task in schedules where they responded more quickly to the arrow, suggesting a high consistency between these two measures. Additionally, motivation prior to blocking in the 10 schedule stages was significantly correlated with mean frustration (r = 0.97, *p* < .001). In schedules where participants reported a higher motivation to complete the task, they also reported more frustration when they were blocked from the reward. For more details, see Figure 3.

### Exploratory analyses on the arrow RTs and confirm RTs

In the analyses above, we assessed the effects of proximity and effort separately, using the same data analysis approach adopted by Yu *et al*. (2014). As an exploratory analysis to assess both effects simultaneously, we analyzed both arrow RTs and confirm RTs using Bayesian mixed-effects models in brms ( Bürkner, 2021). For both analyses, the RT data were first log transformed. We simultaneously included proximity level (from 1 to 4, with a larger value standing for more proximity to a reward) and effort level (from 1 to 4, with a larger value standing for more expended effort) as predictors, with random intercepts and random slopes per participant. Both proximity and effort were modelled as monotonic predictors ( Bürkner & Charpentier, 2018). The Student’s t distribution was used as the likelihood function rather than the default normal distribution, as the latter did not fit the arrow RT data well. Default priors in brms were used. For MCMC sampling, we ran 4 parallel chains with 3000 iterations in the warm-up phase and 5000 iterations in the sampling phase per chain. We inspected the trace plots, R hat values, effective sample sizes and posterior predictive checks to ensure that there were no convergence issues and that the estimates were stable.

For the arrow RT, we observed a negative coefficient for proximity (estimate = -0.017, 95% CI = [-0.024, -0.011]). This indicates that participants responded more quickly in an arrow task when they were closer to the end of a schedule. The coefficient for effort was also negative, estimate = -0.008, 95% CI = [-0.013, -0.004], indicating that participants responded more quickly in an arrow task when they had exerted more effort (i.e., completed more trials) in a schedule so far. For the confirm RT, we observed a different pattern: the coefficient for proximity was positive, estimate = 0.017, 95% CI = [0.001, 0.350], while the coefficient for effort did not differ from 0 credibly, estimate = 0.003, 95% CI = [-0.019, 0.027]. The exploratory analysis thus showed that contrary to the effect of proximity on arrow RTs, participants confirmed an outcome more slowly when the reward blockage was closer to the end of a schedule. This was in contrast to the original study, which did not find any effect on the confirm RT. Note that when we used the same data analysis approach above as in the original study, we also did not observe statistically significant effects of proximity or effort. This discrepancy in results might arise because the exploratory analysis here included proximity and effort simultaneously in one model and analyzed data on each trial (rather than aggregated means in each condition), and thus might have more statistical power to detect an effect. Yu *et al.* did not report or visualize the results on confirm RTs, so it is unclear whether a similar pattern was also present in their data. These exploratory results on confirm RTs were also in line with our finding that participants were more surprised by reward blockage when proximity was high, but expended effort did not influence self-reported surprise. The positive correlation between self-reported surprise and the mean RT of confirming a blocked outcome ( Figure 3) further corroborated the idea that being blocked when getting close to a reward might be a surprising event that slowed responses down.

### facial EMG analyses

**Figure 4.**
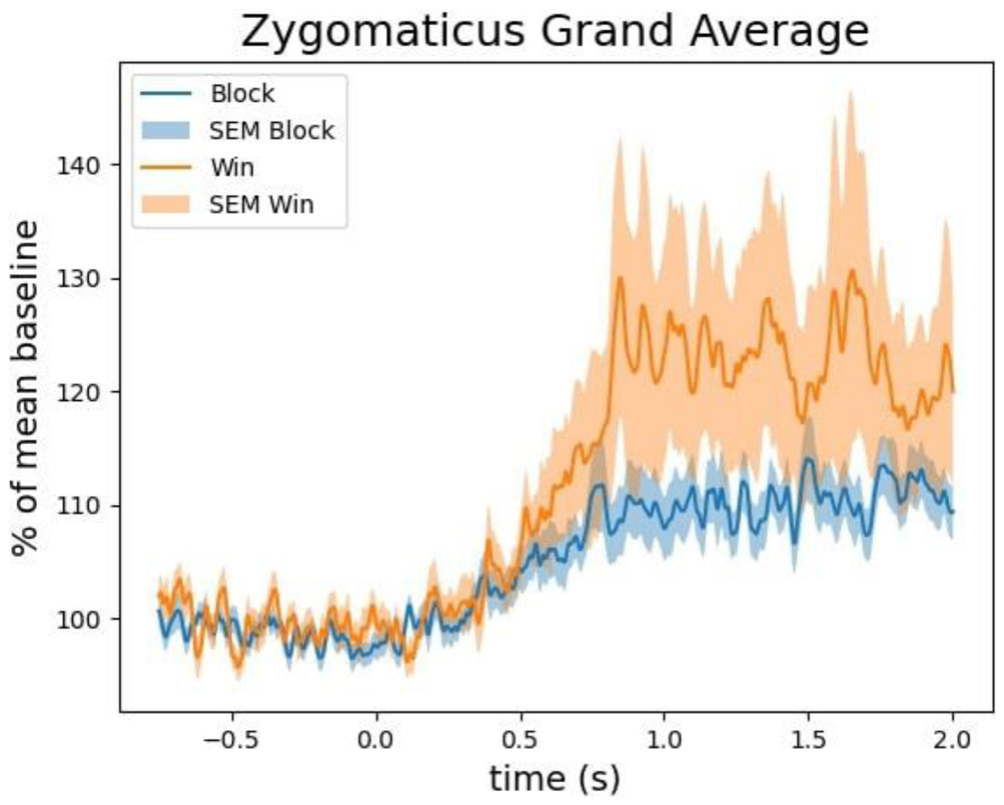
The grand average of the activity over the zygomaticus major as a function of time after the outcome presentation and trial type (win vs. blocked).

**Figure 5.**
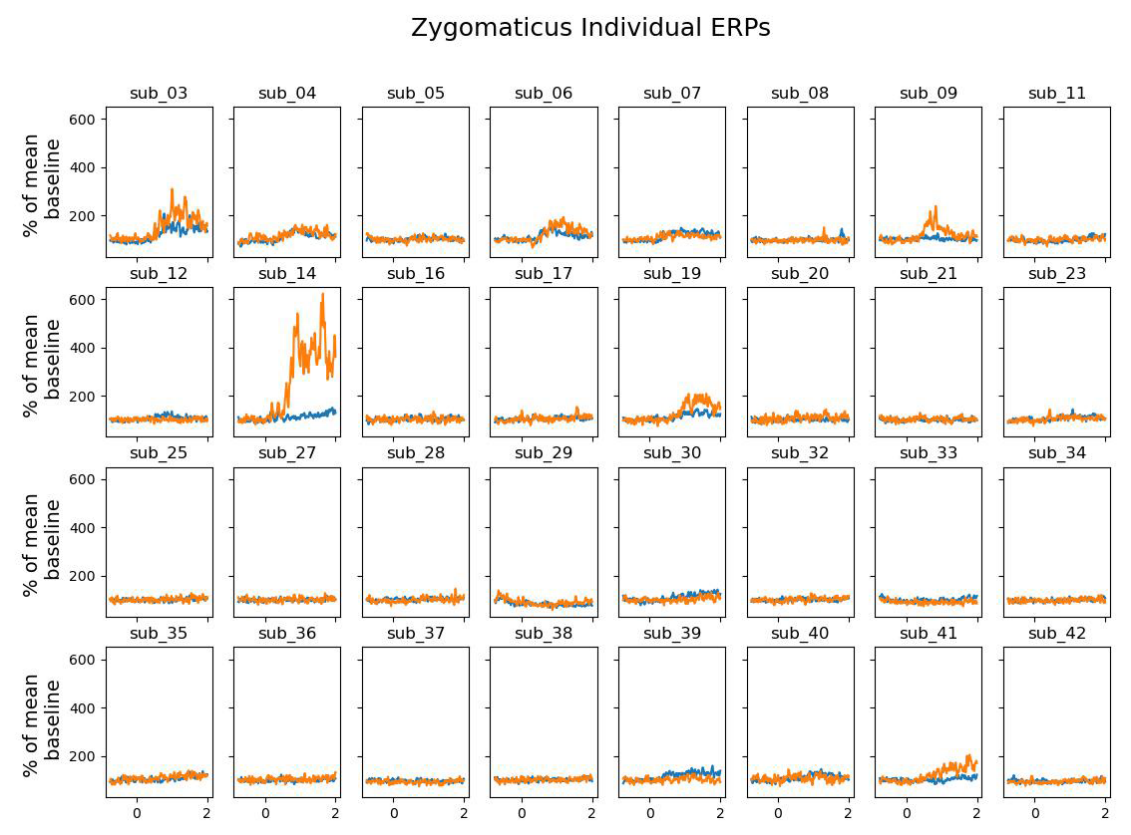
The grand average of the activity over the zygomaticus major as a function of time after the outcome presentation and trial type (win vs. blocked) for each individual.

**Figure 6.**
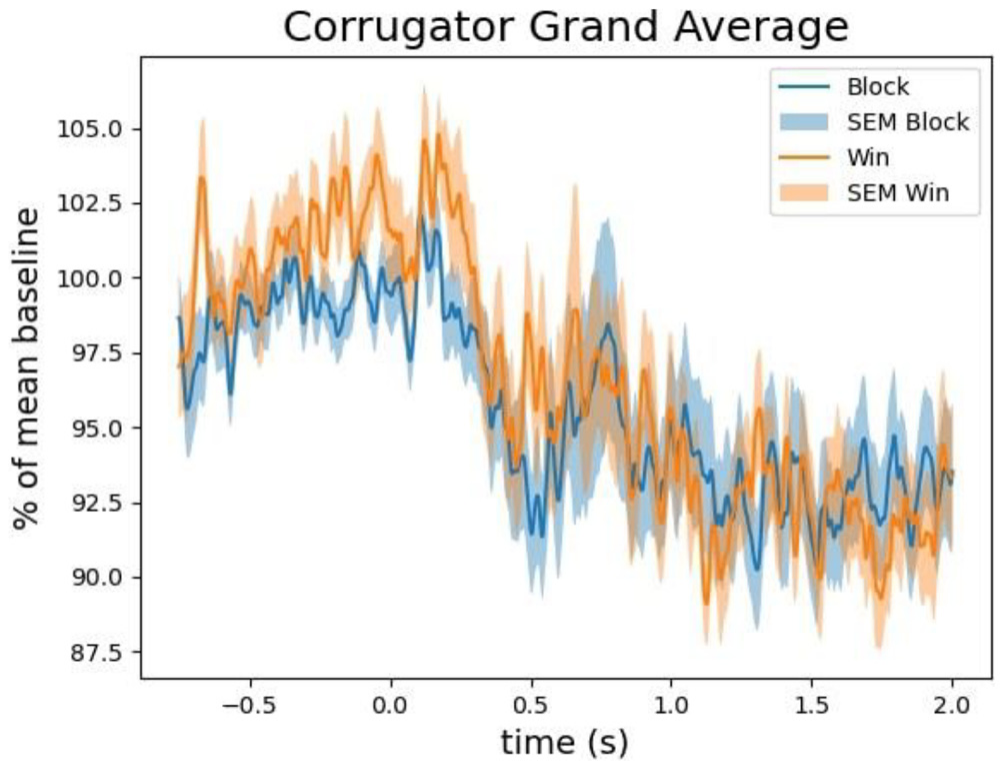
The grand average of the activity over the corrugator supercilii as a function of time after the outcome presentation and trial type (win vs. blocked).

**Figure 7.**
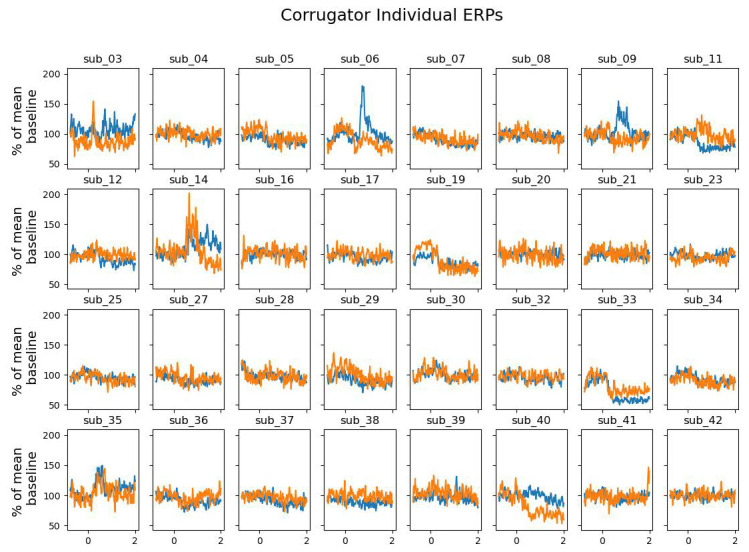
The grand average of the activity over the corrugator supercilii as a function of time after the outcome presentation and trial type (win vs. blocked) for each individual.

We tested whether the zygomaticus major and corrugator supercilii muscles were differentially activated in win versus blocked trials. Being essential for frowning, we expected the corrugator supercilii to be more active in block trials. We expected the zygomaticus major, responsible for smiling, to be more active in win trials. None of our expectations were borne out by the data. Figure 4 shows the grand average activation of the zygomaticus major muscle for win and block trials. While on average, the zygomaticus major is more active on win trials, this difference was not significant (largest cluster, which is the sum of all *t*-values of all time points in the cluster was *t*(sum) = -34 with a *p* = .84). The individual data shown in Figure 5 reveals that this difference was driven by only a small proportion of participants. The corrugator supercilii also did not reveal any differential activity between win and block trials (largest cluster, which is the sum of all *t*-values of all time points in the cluster was was *t*(sum) = 164 with a *p* = .15; see Figure 6 and Figure 7).

## Discussion

Humans and non-human animals often face obstacles that block them from achieving their goals, which can lead to frustration. In the current study, we investigated the influence of goal proximity and expended effort on frustration by trying to replicate and extend the study by Yu *et al*. (2014). For this, participants completed multi-trial schedules in order to obtain a reward. On each trial they had to indicate the direction of arrows by a left or right button press, and had to complete one to four arrow trials successfully to obtain a reward, depending on the schedule. Importantly, they could get blocked at any stage of the schedule, resulting in different levels of goal proximity (i.e., the number of trials that still needed to be completed to obtain the reward) and expended effort (i.e., the number of trials that were already completed). In line with Yu *et al*. (2014), we measured the RT in the arrow task, the RT and force with which participants confirmed the outcome of the current schedule, and self-reported surprise, motivation and frustration for each schedule stage at the end of the experiment. As an extension, we also measured the activity over the corrugator supercilii and the zygomaticus major as online measures of emotional valence. Note that our force measure was most likely underestimated. Thus, we will not discuss any theoretical implications related to these force results and will instead focus on the remaining measures.

In the self-report measures, we were able to replicate Yu *et al*. (2014)’s finding that the closer participants were to the reward and the more effort they had exerted, the more motivation they felt in the arrow task. In line with their self-reported motivation, participants responded faster to the arrows the closer they got to the expected reward and the more effort they had exerted. When they were eventually blocked from a reward, they reported more frustration when getting blocked closer to the reward and with more effort exerted. Using the same data analysis approach as in the original research (i.e., combining the p values from multiple ANOVAs), we similarly observed no effect of proximity and effort on the RT of confirming a blocked outcome. In sum, we were able to replicate the effects (or the absence of the effects) of proximity and effort on arrow RTs and confirm RTs, and self-reported motivation and frustration at the end of the experiment.

Some results differed from those of Yu *et al*. (2014) though. First, participants were more surprised by a blocked outcome when they were getting closer to the end reward, while exerted effort did not influence surprise. This pattern of results was corroborated by the results of an exploratory analysis on confirm RT, in which we found that higher proximity (but not higher effort) increased the RT of confirming an outcome. Furthermore, self-reported surprise also correlated with confirm RT, so that the more surprised participants felt in a schedule, the slower they were in confirming the outcome. Lastly, in the facial EMG data, we found no difference in activation when winning compared to getting blocked, neither in the corrugator supercilii nor in the zygomaticus major.

In general, our replication results seem to be valid despite the underestimation of the force data: the self-report measures show that we were able to induce frustration in participants and that they were generally more motivated to successfully finish the task in later schedule stages. Furthermore, our RT data are likely valid. The mean arrow RTs in the 10 schedules were negatively correlated with the mean self-reported motivation, suggesting that participants indeed responded more quickly when they were more motivated to complete a schedule. The finding that participants got slower in confirming their outcome after they had been blocked at later schedule stages is in contrast to the original study, but in line with the self-reported surprise ratings. In the original study, Yu *et al*. (2014) did not find an effect of surprise. Here however, we found that participants are more surprised the later they got blocked. In the outcome phase, blocking trials were more likely than win trials. However, each schedule contained varying number of trials, and the participants did experience many correct trials on those schedules where they were eventually blocked. From Figure 1, one can compute that the whole design contains 350 correct arrow trials, and 140 ‘blocked’ arrow trials (the probability of being blocked after an arrow trial was therefore around 28%). Those correct responses potentially influenced the participants expectation to win on the next trial. Thus, this surprise might have induced an orienting response ( Notebaert *et al.*, 2009; Wessel, 2018) leading to slower response times in the confirm task after getting blocked at a later schedule stage. In summary, it seems that participants were highly motivated, which was reflected in the response speed as long as they thought they had control over the outcome. Once they got blocked, participants were surprised and took longer to confirm this outcome ( Notebaert *et al*., 2009). Note that other other research using gambling tasks found speeding after reward blockage (see Eben *et al*., 2020; Verbruggen *et al*., 2017). Since earlier research however suggests that perceived control over the outcome plays an important role in whether participants speed up or slow down after sub-optimal outcomes ( Eben *et al*., 2023), we assume that here, participants might have felt in control over the outcome and hence showed an orienting response after being blocked.

Our facial EMG results did not show the expected results. While some participants showed the expected effects most of the participants did not show much activation difference over the corrugator supercilii or the zygomaticus major for wins and blocked trials. However, given that some participants showed more activation of the zygomaticus major than over the corrugator supercilii in the win trials, we assume that fEMG in general seems to be a suitable online measure of emotional valence ( Mauss & Robinson, 2009). Here we assume that the sample size might have been too small to detect reliable differences in psychophysiological measurements. After all, the sample size was calculated based on a behavioral medium effect size.

As already mentioned, we ran into post-hoc problems with our force measurements, presumably because not all participants placed their fingers directly and constantly on top of the sensors. Unfortunately, we have to assume that all force data are underestimated in our experimental set-up, even in participants who do not have 0 values in their force measurement. Note that similar set-ups with sensors on top of keys have been used successfully in previous studies to measure force (e.g., Pickering *et al*., 2020), albeit not in the context of examining ’frustration’. In hindsight, the requirement for participants to keep their fingers constantly on the sensors (and not moving their fingers) may be inappropriate in the current study, as the ’frustration’ triggered by blocked reward may lead participants to move their fingers despite the instructions to not do so. This may be the reason why the force measures with our custom box were invalid. Alternative ways of measuring force exist, such as by attaching buttons to a leaf spring, and measuring the force applied via strain gauges (e.g., Ko *et al*., 2012). In such a set-up, force is determined by measuring how much the leaf spring is bent (which in turn is measured by strain gauges). There will therefore be no requirement for participants to keep their finger locations constant on the keys. While we initially experimented with this idea, we eventually decided to not use such a set-up, because the tactile feedback of keys being pressed down was much less clear in this set-up than in our adopted set-up. Since we used rigged feedback to introduce blocked reward, such uncertainty in whether a key had indeed been pressed down or not might lead participants to adjust their subsequent responses, such as pressing keys harder to make sure that a response was registered. To reduce this potential influence, we therefore did not use this alternative set-up in our study. Yu *et al*. (2014) did not provide technical details on the custom box they used, so it is unclear whether these differences in the boxes may explain the different results.

Despite the force measurements being invalid, in light of a continued publication bias in psychological research ( Scheel *et al*., 2021), we would like to follow the call of Lakens and Ensinck (2024) and make this research available, hoping it might be helpful for others who want to use the measure of response force or try to replicate the original study (see Pennington *et al*., 2023, for a similar take on publishing results that might not answer the research question).

In conclusion, although the self-report measures suggest that frustration was induced in our participants by blocking them from an expected reward, we were unable to replicate all the behavioral findings of Yu *et al.* (2014). As in the original study, our RT data showed participants sped up in the arrow task the closer they got to the reward. Once they were blocked, however participants seemed to be surprised about the outcome and confirmed the outcome with a slowed response. These results were in contrast to the original findings, which did not find any effects of surprise or confirm RT. Given that our current results are not fully in line with the original study, yet plausible, we call for further investigations of frustration and the effects of goal proximity and exerted effort on frustration.

## Ethics and consent

The research was approved by the Ethics committee of the Faculty of Psychology and Educational Sciences of Ghent University (ethical approval number 2022–135 approved 17/01/2023). Participants provided written informed consent before starting the experiment.

## Data Availability

Open Science Framework: “Escalating frustration - A replication attempt and extension of Yu *et al*. (2014)”, DOI: 10.17605/OSF.IO/RMH2D ( Eben *et al*., 2024). This project contains the following underlying data: Code – analysis code and experimental code including supporting documents Data – raw and processed data. Raw: One data file per participant for demographics, force, behavioral data, self-report ratings, raw force, event trigger journals and fEMG raw data. Processed: For details please see data ducmentation in the documentation folder. Documentation - Three .md documents containing detailed documentation about data, analysis and the general experimental procedure and conditions. A folder with results overview files including extended analyses and a folder with supporting documents such as the lab notes, the handedness questionnaire and the checklist used for our fEMG testibg procedure. Checklist for “Escalating frustration - A replication attempt and extension of Yu *et al*. (2014)”, https://osf.io/vsfe2 Creative Commons Attribution 4.0 International Public License
