# Supplementary material for: Escalating frustration - A replication attempt and extension of Yu et al. (2014)
Source: Open Res Eur. 2025 Apr 10;4:135. Originally published 2024 Jul 9. [Version 2] doi: 10.12688/openreseurope.17749.2 (PMC12498315; doi:10.12688/openreseurope.17749.2)
Supplement: Peer review history

# Reviewer report: Reviewer response for version 2

**DOI:** 10.21956/openreseurope.21800.r53210

**Published:** 2025-05-10

**Authors:** Aijaz A. Naik

**Affiliations:** 1 National Institute of Mental Health (NIMH), Bethesda, USA

**Major Comments**

**1. Incomplete Theoretical Framework and Literature Integration**

 The manuscript appears to present only a truncated introduction, referencing amendments from a previous version without a full narrative arc. While key citations such as Devine et al. (2024), Katzir et al. (2020), and Amsel (1958) are mentioned, the broader theoretical context of frustration, emotional regulation, and facial EMG remains underdeveloped.

- A complete introduction is essential to establish the theoretical relevance of facial EMG in the context of frustration research.
- The authors should clearly articulate the conceptual gaps, particularly regarding the multimodal relationship between behavioral responses, self-reported affect, and physiological data.
- The manuscript would benefit from integrating relevant models of emotion (e.g., the valence-arousal framework or frustration-aggression theory) to ground their hypotheses.

**Suggestions:**

- Situate the current study within classic and contemporary frustration paradigms.
- Discuss how the EMG findings extend or replicate known effects (e.g., zygomaticus inhibition or corrugator activation in negative affect).
- Explicitly define the novelty of the current approach—what does this study reveal that has not been addressed by prior work?

**2. Unclear Research Questions and Hypotheses**

 While the methods and results are outlined, the absence of clearly stated research questions or a hypothesis framework makes it difficult to evaluate the logical flow of the study.

- Are the authors predicting stronger corrugator activation and zygomaticus suppression in blocked (vs. win) trials?
- How are these patterns expected to correlate with self-report measures?
- Do the authors expect individual differences in these patterns, and if so, are they theory-driven?

**Recommendations:**

- Clearly state specific, testable hypotheses.
- Justify predictions based on past literature, especially within affective neuroscience or psychophysiology.

**3. Methodological Gaps in Experimental Design**

 While the general paradigm appears to involve alternating win and blocked reward trials, the manuscript lacks critical details:

- **Operationalization of frustration:** How is the blocked outcome manipulated to induce frustration?
- **Force data omission:** The authors state the force data was invalid but fail to explain why or how this impacts interpretation.

**Concerns:**

- Was the experimental task pre-registered?
- How was the trial order counterbalanced, if at all?

**4. Bayesian Modeling and Statistical Transparency**

 The manuscript indicates a shift from ANOVA to multilevel Bayesian models using brms, yet reporting is vague and lacking transparency.

**Required clarifications:**

- Justification for choosing Bayesian multilevel modeling beyond general statements.
- Clear specification of model structure: priors, fixed and random effects, convergence diagnostics (e.g., R-hat, ESS).
- Report of model fit indices (e.g., WAIC, LOO) if applicable.
- Presentation of posterior distributions, credible intervals, and interpretation of effect sizes.

**5. Missing or Severely Truncated Discussion**

 The manuscript lacks a robust discussion section. As a result:

- The theoretical implications of the findings remain unclear.
- There is no reflection on how the results support or challenge existing frustration theories.
- Alignment (or misalignment) between self-report and EMG data is not addressed.
- Limitations and future directions are not discussed.

**Suggestions:**

- Reconnect the findings with the introduction and hypotheses.
- Discuss whether EMG data serve as valid indices of affective frustration.
- Address real-world implications or extensions to related domains (e.g., decision-making, cognitive control, psychopathology).

**Minor Comments**

**1. Figures and Captions**

- **Figure 1** does not clearly explain what is being shown; a timeline or schematic of the task would be more informative.
- **Figure 2** has panels a–f but lacks sufficient explanation of each panel and the relevant results.
- **Figures 5–7** need:
  - Y-axis units
  - Directionality of effect explained (e.g., does higher corrugator activity mean more negative affect?)
  - Participant identifiers (or consistent ordering) for better cross-referencing.

**2. Statistical Reporting Inconsistencies**

- Table 1 includes inconsistently reported p-values (some with <, others exact).
- The statistical tests corresponding to the reported F-values are not described.
- Effect sizes (e.g., η², Cohen’s d) are missing.
- Multilevel model results are not clearly summarized with parameter estimates.

**4. Terminology and Accessibility**

- Briefly define terms like “zygomaticus major” and “corrugator supercilii” for broader audiences.
- Avoid jargon like “post-loss speeding” without first defining it.

If applicable, is the statistical analysis and its interpretation appropriate?

Partly

Is the study design appropriate and is the work technically sound?

Partly

Is the work clearly and accurately presented and does it engage with the current literature?

Partly

Are the conclusions drawn adequately supported by the results?

Partly

Are sufficient details of methods and analysis provided to allow replication by others?

Partly

Are all the source data and materials underlying the results available?

Partly

Reviewer Expertise:

Neuroscience, Plasticity, Engrams, Frustration-non-reward, circuits,

I confirm that I have read this submission and believe that I have an appropriate level of expertise to confirm that it is of an acceptable scientific standard, however I have significant reservations, as outlined above.

# Reviewer report: Reviewer response for version 2

**DOI:** 10.21956/openreseurope.21800.r53044

**Published:** 2025-05-09

**Authors:** Sean Devine

**Affiliations:** 1 Department of Psychology, McGill University, Montreal, Québec, Canada

I am satisfied with the authors' response to my comments and commend them on their contribution to the field.

If applicable, is the statistical analysis and its interpretation appropriate?

Yes

Is the study design appropriate and is the work technically sound?

Yes

Is the work clearly and accurately presented and does it engage with the current literature?

Yes

Are the conclusions drawn adequately supported by the results?

Partly

Are sufficient details of methods and analysis provided to allow replication by others?

Yes

Are all the source data and materials underlying the results available?

Yes

Reviewer Expertise:

Cognitive psychology, cognitive effort, goal-gradient effects, EMG analysis, computational modeling, multilevel modeling

I confirm that I have read this submission and believe that I have an appropriate level of expertise to confirm that it is of an acceptable scientific standard.

# Reviewer report: Reviewer response for version 2

**DOI:** 10.21956/openreseurope.21800.r53045

**Published:** 2025-05-07

**Authors:** Sebastian Korb

**Affiliations:** 1 Psychology, University of Essex (Ringgold ID: 2591), Colchester, England, UK; 2 Psychology, University of Vienna, Vienna, Austria

I am pleased with the changes made to the manuscript, which in its latest version has much improved. I especially found useful the new figure 2 and discussion on which of the original findings had been replicated and which ones had not. I have no further comments and commend the authors for a nice piece of work.

If applicable, is the statistical analysis and its interpretation appropriate?

Yes

Is the study design appropriate and is the work technically sound?

Yes

Is the work clearly and accurately presented and does it engage with the current literature?

Yes

Are the conclusions drawn adequately supported by the results?

Yes

Are sufficient details of methods and analysis provided to allow replication by others?

Yes

Are all the source data and materials underlying the results available?

Yes

Reviewer Expertise:

emotion, facial expressions, embodiment, facial EMG, EEG

I confirm that I have read this submission and believe that I have an appropriate level of expertise to confirm that it is of an acceptable scientific standard.

# Reviewer report: Reviewer response for version 1

**DOI:** 10.21956/openreseurope.19188.r50620

**Published:** 2025-02-21

**Authors:** Sebastian Korb

**Affiliations:** 1 Psychology, University of Essex (Ringgold ID: 2591), Colchester, England, UK; 2 Psychology, University of Vienna, Vienna, Austria

The manuscript describes a replication attempt of a study by Yu et al. (2014), who had shown that the more participants have already put effort into receiving a reward, and the closer they are to getting it, the more they become frustrated when blocked from receiving the announced reward, resulting in increased response force in the next trials. The same methods were used as in the original study, but in addition facial EMG was acquired from the main smiling (zygomaticus major) and frowning (corrugator supercilii) muscles. The force measurement was faulty and therefore not included (but see supplementary materials), and the facial EMG did not result in significant effects. Other than that, the main findings by Yu et al. (2014) were replicated, although participants reported higher levels of surprise about blocked trials, compared to the original study.

 The methods are sound, and the manuscript is well written. I mainly have minor comments (see below), but also struggle with the authors’ conclusion that participants were surprised by the ‘blocking’ trials. This interpretation seems unlikely to me, based on the fact that blocking trials were 2 times more likely (n = 140) than win trials (n = 70). Also, surprise was only measured retrospectively, at the end of the experiment (it is thus memory about feeling surprised).

 # Abstract

 _clarify that the following results were unexpected: “participants were slower in confirming an outcome when they were blocked closer to the reward”

 _the phrase “confirm RT” is unclear to the naïve reader

 # Introduction & Methods

 _p. 4: “In each schedule, some parts of the coin were already given to participants for ’free’, so the number of ’trials’ varied from one (i.e., only one quarter of the coin was missing) to four (i.e., the whole coin was missing).” - this sentence suggests that the experiment included schedules in which all parts of the coin were missing, but Figure 1 suggests otherwise. Similarly, text in the first paragraph on page 6 is not compatible with this scenario (“Depending on how many trials needed to be completed, parts of the coin were visible as well (e.g., if participants had to complete three trials, a quarter of the coin was already visible).” – how can parts of the coin be visible in schedules with all coin quarters missing?). Please clarify or correct if necessary.

 _p. 6-7 explains that participants were told that at the end of the experiment “the program would randomly pick 10 schedules. For each win outcome, they would receive 2 euros. For each blocked outcome, they would receive no extra money. […] the results of these 10 selected schedules would be added up, to determine their final bonus (with 10 euros as the maximum)”. If I understand this correctly, participants were first told they could potentially earn 20 euros (10 win schedules x 2 euros), but then that they would maximally receive half of that? If that is correct, I would find it rather disappointing (and confusing) for participants.

 _p. 7: the word “filter” is missing after “We applied a 20-200 Hz band pass”

 _p.7: trials with an RT of 0 ms were removed from analyses, but how come trials with very fast (i.e. < 100 or 200 ms) RTs were kept in the analyses? It would also seem implausible to answer that fast.

 # Results

 _Figure 2: please number the panels (also given that the caption refers to the 5 panels by number), improve the y-axis label in panel 1 (change to “confirm RT (ms)”), either make all x-axis ticks the same or explain why the differ (some include blocked trials, some do not), and clarify which of these analyses are on win/block trials only or on both types of trials. Finally, is it possible to indicate significant effects (e.g. with a star)?

 _Figure 4: please add a ribbon around the EMG lines to show some measure of variance (e.g. SEM or 95% CI)

 _Figures 5 and 7 are interesting but not necessary in the main text, I would move them to the supplementary materials

 _no significant effects were found in the facial EMG. However, this could be due to the somewhat unusual analysis, which included all time points and then applied a correction for multiple comparisons. Instead, you could average over time windows of 250 or 500 ms, and then include the factor time (with 4 or 8 levels).

 _p10: missing ‘s’ after ‘participant’: “For this, participant completed multi-trial schedules”

If applicable, is the statistical analysis and its interpretation appropriate?

Yes

Is the study design appropriate and is the work technically sound?

Yes

Is the work clearly and accurately presented and does it engage with the current literature?

Yes

Are the conclusions drawn adequately supported by the results?

Yes

Are sufficient details of methods and analysis provided to allow replication by others?

Yes

Are all the source data and materials underlying the results available?

Yes

Reviewer Expertise:

emotion, facial expressions, embodiment, facial EMG, EEG

I confirm that I have read this submission and believe that I have an appropriate level of expertise to confirm that it is of an acceptable scientific standard, however I have significant reservations, as outlined above.

# Response

**Published:** 2025-04-02

**Authors:** Charlotte Eben

**Affiliations:** Department Psychology, University of Cologne, Cologne, North Rhine-Westphalia, Germany

1. I mainly have minor comments (see below), but also struggle with the authors’ conclusion that participants were surprised by the ‘blocking’ trials. This interpretation seems unlikely to me, based on the fact that blocking trials were 2 times more likely (n = 140) than win trials (n = 70). Also, surprise was only measured retrospectively, at the end of the experiment (it is thus memory about feeling surprised).

 
**Reply**: *We have added this to the discussion about the results on the surprise ratings (p.18):* “In the outcome phase, blocking trials were more likely than win trials. However, each schedule contained varying number of trials, and the participants did experience many correct trials on those schedules where they were eventually blocked. From Figure 1, one can compute that the whole design contains 350 correct arrow trials, and 140 ‘blocked’ arrow trials (the probability of being blocked after an arrow trial was therefore around 28%). This probability of correct responses potentially influenced the participants expectation to win on the next trial. Thus, this surprise might have induced an orienting response (Notebaert et al., 2009; Wessel, 2018) leading to slower response times in the confirm task after getting blocked at a later schedule stage.”  

1.  _clarify that the following results were unexpected: “participants were slower in confirming an outcome when they were blocked closer to the reward”

 
**Reply**: *We have done that.*  

1.  _the phrase “confirm RT” is unclear to the naïve reader

 
**Reply**: *We have changed the wording to ‘the time taken to confirm the outcome’.*  

1.  _p. 4: “In each schedule, some parts of the coin were already given to participants for ’free’, so the number of ’trials’ varied from one (i.e., only one quarter of the coin was missing) to four (i.e., the whole coin was missing).” - this sentence suggests that the experiment included schedules in which all parts of the coin were missing, but Figure 1 suggests otherwise. Similarly, text in the first paragraph on page 6 is not compatible with this scenario (“Depending on how many trials needed to be completed, parts of the coin were visible as well (e.g., if participants had to complete three trials, a quarter of the coin was already visible).” – how can parts of the coin be visible in schedules with all coin quarters missing?). Please clarify or correct if necessary.

 
**Reply**: *All schedules in which participants had to complete four trials to receive the coin started without any part of the coin given. We have now revised the texts throughout the manuscript to make this clearer.* *Figure 1 only indicates the outcome trials, but not the progression of trials within a schedule. We have now also clarified this in the figure caption. For further information, see the video of the trial procedure on OSF:* *https://osf.io/z28b4* *.*  

1.  _p. 6-7 explains that participants were told that at the end of the experiment “the program would randomly pick 10 schedules. For each win outcome, they would receive 2 euros. For each blocked outcome, they would receive no extra money. […] the results of these 10 selected schedules would be added up, to determine their final bonus (with 10 euros as the maximum)”. If I understand this correctly, participants were first told they could potentially earn 20 euros (10 win schedules x 2 euros), but then that they would maximally receive half of that? If that is correct, I would find it rather disappointing (and confusing) for participants.

 
**Reply**: *Indeed, participants were told that they could receive extra bonus and that this bonus would be the added results of the 10 chosen trials with 2€ per win. At the same time, they were informed that the maximum payout was 10€. They received this information before the experiment started and it is a common way of keeping the payout somewhat equal in gambling like tasks where the experimenter does not have control over the outcome. In this case, all participants received the 10€ maximum payout in addition to the 15€ reimbursement for their participation. None of the participants complained about this payout structure and in fact, most of them were pleasantly surprised that they would leave the experiment with 10€ extra.*  

1.  _p. 7: the word “filter” is missing after “We applied a 20-200 Hz band pass”

 
**Reply**: *We have corrected this.*  

1. _p.7: trials with an RT of 0 ms were removed from analyses, but how come trials with very fast (i.e. < 100 or 200 ms) RTs were kept in the analyses? It would also seem implausible to answer that fast.

 
**Reply**: *Yu et al. (2014) did not report any exclusion criterion for RT analyses. To be consistent with their analysis approach, we therefore also did not implement any exclusion criterion, except for RTs of 0 which might indicate premature responses that were initiated before the stimulus presentation. However, considering this comment, we repeated the ANOVA analysis as in the original analysis, but with RTs below 200 ms excluded. For the confirmation RT, this led to the exclusion of 0.03% of the trials. For the arrow RT, this led to the exclusion of 0.18% of the trials. Importantly, all results reported in the manuscript remain the same. Since (1) only a small number of trials had an RT below 200ms, (2) the results remain the same with and without excluding RTs below 200ms, and (3) Yu and colleagues did not use such an exclusion criterion, we decide to report the results without excluding RTs below 200 ms, as they currently are, in the manuscript.*  

1.  _Figure 2: please number the panels (also given that the caption refers to the 5 panels by number), improve the y-axis label in panel 1 (change to “confirm RT (ms)”), either make all x-axis ticks the same or explain why the differ (some include blocked trials, some do not), and clarify which of these analyses are on win/block trials only or on both types of trials. Finally, is it possible to indicate significant effects (e.g. with a star)?

 
**Reply**: *We have added letters to the panels, improved the y-axis of the confirm RT and added explanations to the schedule stages. As explained on p. 9, we only included blocked trials in the analyses (and correct trials for the arrow task). We are however not able to add indications of significance since the effects we looked at are not reflected in the descriptives (effort vs. proximity effect p. 9/10 for further explanation). We have added the original plots for better comparison.*  

1. _Figure 4: please add a ribbon around the EMG lines to show some measure of variance (e.g. SEM or 95% CI)

**Reply:** *We have added 95% CI as a ribbon around the EMG lines in Figure 4 and Figure 6.*  

1.  _Figures 5 and 7 are interesting but not necessary in the main text, I would move them to the supplementary materials

 
**Reply**: *We think it is important to highlight the substantial differences across participants, which is often not shown when only group averages are presented. We also refer to these individual differences in the manuscript when discussing the fEMG results. For these reasons, we think it is more convenient to have Figures 5 and 7 in the manuscript.*  

1.  _no significant effects were found in the facial EMG. However, this could be due to the somewhat unusual analysis, which included all time points and then applied a correction for multiple comparisons. Instead, you could average over time windows of 250 or 500 ms, and then include the factor time (with 4 or 8 levels).

 
**Reply**: *Thank you for this suggestion. When analyzing the fEMG data, we followed an analysis approach that is often used to analyze EEG data. Although not pre-registered, we decided on this approach before looking at the data. While the proposed analysis can be a viable alternative, the selection of time windows (250, 500, or other durations) can be somewhat arbitrary. More importantly, we are concerned that trying out an alternative analysis because our original analysis did not yield significant results, may come close to “p-hacking”. Furthermore, Figures 5 and 7 show large variations across participants. Even if the proposed analysis shows significant results, these results should still be treated with caution.* *For all these reasons, we would like to transparently report the results from our originally planned analysis.*    

1. _p10: missing ‘s’ after ‘participant’: “For this, participant completed multi-trial schedules”

   
**Reply**: *We have corrected this.*

# Reviewer report: Reviewer response for version 1

**DOI:** 10.21956/openreseurope.19188.r42232

**Published:** 2024-07-19

**Authors:** Sean Devine

**Affiliations:** 1 Department of Psychology, McGill University, Montreal, Québec, Canada

**Overall appraisal ---------------------**

 In this paper, Eben and colleagues attempt a replication and extension of Yu et al. (2014). Originally, Yu and colleagues found that self-reported ratings of frustration, motivation, response time, and pressing force varied as a function of goal proximity in a rewarded computerized “blocking” experiment. Eben et al. successfully replicate Yu et al.’s procedure and find in some cases similar results and in others non-consistent results.

 Overall, this paper was well-written and approached the replication with good methods and a clear rationale. I especially appreciated the transparency with which the authors reported their findings and described the technical limitations they ran into. As a replication of a study that’s been cited over 100 times, I think this paper succeeds in providing additional information about the reliability and size of the original effects.

 Below I detail some comments that I think can help further improve this paper.

**Main comments ---------------------**

  

1. I hate to be the reviewer who requests to cite his own work, but in this case I think it is appropriate. In the introduction, the authors discuss both recent and classical work on the goal-gradient hypothesis but neglect the burgeoning literature on goal-gradients in cognitive effort expenditure (Katzir et al., 2020; Emanuel et al., 2022; Devine et al., 2020; 2024). This work seems especially relevant since the current task leverages an attentional task (the arrow task) not too dissimilar from the one in Devine et al. (2024), where we found similar speeding proximity effects. So, at the least, it might be useful to put Yu et al.’s (2014) results (and the current replication) in conversation with some more recent work exploring these proximity effects in psychology. Moreover, the authors also discuss past work which has used EMG to measure affect, but do not discuss more recent work specifically linking EMG activity to cognitive effort exertion (Devine et al., 2024; Berger et al., 2020). This seems like something that would be directly relevant to the current discussion and justification of EMG in this context. I leave it up to the authors if they choose to include this work and they should not feel compelled to do so just because some of this work is mine, as the reviewer’s. I just think some of it is particularly relevant for the topic at hand.
2. One key element which I found unclear throughout the paper was *which *effects Eben and colleagues were aiming to replicate. In their Discussion they state: “Our RT data showed participants sped up in the arrow task the closer they got to the reward. Once they were blocked, participants seemed to be surprised about the outcome and confirmed the outcome with a slowed response [...O]ur current results are not in line with the original study” (p. 14). Yet, in reading Yu et al. (2014), they specifically de-emphasize the confirm-RTs in their analysis: “[Participants] were told that “Confirming the outcome is not a speeded task, and that you have plenty of time (4 sec) to make this response” (p. 167). Further, only RTs to arrows (not confirm-RTs) are reported in Yu et al.’s main text figures and tables. In fact, apart from listing average confirm-RTs, I am not sure where Yu et al. report confirm-RT results whatsoever. Thus, I am not entirely sure what to make of the discussed discrepancy in confirm-RTs. In any case, I am not sure it is correct to position the current replication as strongly inconsistent with the original findings—especially when the majority of behavioral and self-report measures do seem consistent. Perhaps the authors could further clarify their view on the importance of confirm-RTs to the original findings.
3. Regarding statistical power, I think there is a bit more work to be done here. Firstly, I was not clear how the authors computed power for their analyses. They report a benchmark Cohen’s d (0.5) which they are able to detect with 80% power. But both their replication analyses (ANOVAs) and exploratory analyses (Bayesian MLMs) do not have effect sizes on this metrics. So perhaps a conversion was done?
  Leaving this question aside, the authors’ rightfully highlight that using Yu et al.’s ANOVA-based analysis, they too fail to find a significant confirm-RT effect (replicating the original null finding). However, when using a multilevel approach, this proximity effect on confirm-RTs emerges. I wholly agree that the multilevel approach is the superior one to the ANOVA-based analysis. However, calculating power in an MLM context is quite different than doing so for ANOVA and usually requires simulation. If indeed the MLM approach afforded increased power over the inferior ANOVA approach, then it could be that the original Yu et al. study was simply underpowered to detect the proximity effect on confirm-RTs. Again, it is hard to judge since, from what I can see, Yu et al. do not report or visualize any confirm-RT data in their original publication. But if this is indeed just a power issue (e.g., Yu et al also found numerical increases in confirm-RTs as a function of proximity, but which failed to achieve significance under an ANOVA approach), this would be a helpful piece of information for explaining the discrepancy in results.
4. Going beyond replication-by-significance, it would be interesting to see a brief discussion of relative effect sizes. It is difficult to compare right now because arrow-RT effects are reported by ANOVA in Yu et al. but by MLM in Eben et al. Nevertheless, eyeballing Figure 2 in Eben et al. against Figure 3 in Yu et al., the RT and self-report effects seem remarkably similar (e.g., a roughly ~40ms difference from 1/4 to 4/4 schedules in both samples). At the most, something like a small-telescopes approach could be used (Simonsohn, 2015), but even just a qualitative comparison of the relative effect sizes—particularly for the confirm-RTs (see power issue from comment #3 above)—could be very informative.
5. Some more details about the fEMG analysis would be helpful. What preprocessing steps were taken? Were signals bandpassed filtered, and if so at what frequency range? Were they rectified and low-pass filtered? (see van Boxtel, 2010).  Some of these cleaning steps may help more subtle effects emerge, especially under conditions of potentially low power as the authors highlight on p. 13.
  Additionally, here the authors adopted a grand-mean ERP approach for computing fEMG signals (p. 8). Instead, the authors could consider a single-trial approach, in which a summary value for each muscle is computed per trial per subject (e.g., the percent change in maximal constriction from baseline). This approach yields a single scalar summary value per trial and could mitigate power issues by keeping more data in the analysis and permitting a multilevel analysis similar to their RT analysis (something like: “cor ~ mo(proximity) + mo(effort) + ( mo(proximity) + mo(effort) | id)”). The downside to this approach is that it precludes a timecourse analysis, but as far as I understand, the timecourse of the EMG signal is less of interest than the comparison of experimental conditions in this case.  See Devine et al. (2023) for an example of this approach.

**Minor comments ---------------------**

  

1. Interestingly, the blocking procedure used here closely mirrors that used in classical experiments on goal-gradients in rats (Brown, 1948). I am not sure if this was an inspiration or not for the original design, but if not, it is an interesting coincidence.
2. I very much liked the video of the experiment uploaded to OSF! More researchers should do this—it makes evaluating the methodology much easier.
3. Though Yu et al also did not analyze accuracy rates, I wonder if proximity or effort meaningfully influenced accuracy rates in this task. Or was accuracy too high and invariable for a simple task as this?

**References ---------------------**

 Berger, A., Mitschke, V., Dignath, D., Eder, A., & van Steenbergen, H. (2020). The face of control: Corrugator supercilii tracks aversive conflict signals in the service of adaptive cognitive control. *Psychophysiology*, *57*(4), e13524.

 Brown, J. S. (1948). Gradients of approach and avoidance responses and their relation to level of motivation. *Journal of comparative and physiological psychology*, *41*(6), 450.

 Devine, S., & Otto, A. R. (2022). Information about task progress modulates cognitive demand avoidance. *Cognition*, *225*, 105107.

 Devine, S., Roy, M., Beierholm, U., & Otto, A. R. (2024). Proximity to rewards modulates parameters of effortful control exertion. *Journal of Experimental Psychology: General*.

 Devine, S., Vassena, E., & Otto, A. R. (2023). More than a feeling: physiological measures of affect index the integration of effort costs and rewards during anticipatory effort evaluation. *Cognitive, Affective, & Behavioral Neuroscience*, *23*(4), 1129-1140.

 Emanuel, A., Katzir, M., & Liberman, N. (2022). Why do people increase effort near a deadline? An opportunity-cost model of goal gradients. *Journal of Experimental Psychology: General*, *151*(11), 2910.

 Katzir, M., Emanuel, A., & Liberman, N. (2020). Cognitive performance is enhanced if one knows when the task will end. *Cognition*, *197*, 104189.

 Simonsohn, U. (2015). Small telescopes: Detectability and the evaluation of replication results. *Psychological science*, *26*(5), 559-569.

 Van Boxtel, A. (2010, August). Facial EMG as a tool for inferring affective states. In *Proceedings of measuring behavior* (Vol. 2010).

If applicable, is the statistical analysis and its interpretation appropriate?

Yes

Is the study design appropriate and is the work technically sound?

Yes

Is the work clearly and accurately presented and does it engage with the current literature?

Yes

Are the conclusions drawn adequately supported by the results?

Partly

Are sufficient details of methods and analysis provided to allow replication by others?

Yes

Are all the source data and materials underlying the results available?

Yes

Reviewer Expertise:

Cognitive psychology, cognitive effort, goal-gradient effects, EMG analysis, computational modeling, multilevel modeling

I confirm that I have read this submission and believe that I have an appropriate level of expertise to confirm that it is of an acceptable scientific standard, however I have significant reservations, as outlined above.

## References

[1] : The face of control: Corrugator supercilii tracks aversive conflict signals in the service of adaptive cognitive control. *Psychophysiology* .2020;57(4) : 10.1111/psyp.13524 e13524 10.1111/psyp.13524 31930536 PMC7079141

[2] : Gradients of approach and avoidance responses and their relation to level of motivation. *J Comp Physiol Psychol* .1948;41(6) : 10.1037/h0055463 450-65 10.1037/h0055463 18102682

[3] : Proximity to rewards modulates parameters of effortful control exertion. *J Exp Psychol Gen* .2024;153(5) : 10.1037/xge0001561 1257-1267 10.1037/xge0001561 38451699

[4] : More than a feeling: physiological measures of affect index the integration of effort costs and rewards during anticipatory effort evaluation. *Cogn Affect Behav Neurosci* .2023;23(4) : 10.3758/s13415-023-01095-3 1129-1140 10.3758/s13415-023-01095-3 37059875

[5] : Why do people increase effort near a deadline? An opportunity-cost model of goal gradients. *J Exp Psychol Gen* .2022;151(11) : 10.1037/xge0001218 2910-2926 10.1037/xge0001218 35511565

[6] : Cognitive performance is enhanced if one knows when the task will end. *Cognition* .2020;197: 10.1016/j.cognition.2020.104189 104189 10.1016/j.cognition.2020.104189 31978813

[7] : Small telescopes: detectability and the evaluation of replication results. *Psychol Sci* .2015;26(5) : 10.1177/0956797614567341 559-69 10.1177/0956797614567341 25800521

[8] : Crosstalk in Facial EMG and Its Reduction Using ICA. *Sensors (Basel)* .2023;23(5) : 10.3390/s23052720 10.3390/s23052720 36904924 PMC10007323

# Response

**Published:** 2025-04-02

**Authors:** Charlotte Eben

**Affiliations:** Department Psychology, University of Cologne, Cologne, North Rhine-Westphalia, Germany

1. I hate to be the reviewer who requests to cite his own work, but in this case I think it is appropriate. In the introduction, the authors discuss both recent and classical work on the goal-gradient hypothesis but neglect the burgeoning literature on goal-gradients in cognitive effort expenditure (Katzir et al., 2020; Emanuel et al., 2022; Devine et al., 2020; 2024). This work seems especially relevant since the current task leverages an attentional task (the arrow task) not too dissimilar from the one in Devine et al. (2024), where we found similar speeding proximity effects. So, at the least, it might be useful to put Yu et al.’s (2014) results (and the current replication) in conversation with some more recent work exploring these proximity effects in psychology. Moreover, the authors also discuss past work which has used EMG to measure affect, but do not discuss more recent work specifically linking EMG activity to cognitive effort exertion (Devine et al., 2024; Berger et al., 2020). This seems like something that would be directly relevant to the current discussion and justification of EMG in this context. I leave it up to the authors if they choose to include this work and they should not feel compelled to do so just because some of this work is mine, as the reviewer’s. I just think some of it is particularly relevant for the topic at hand.

**Reply:** *Thank you for this suggestion. We have added some of your suggested articles to our introduction of goal gradient on p.4.* “Many goal pursuit activities require individuals to carry out multiple successive actions to obtain a reward. Accordingly, individuals may fail at any stage of the goal pursuit process, which in turn may influence the magnitude of the ’frustration’ effect triggered by reward omission or blockage. As individuals make progress in their goal pursuit, their motivation to obtain the goal generally increases (i.e., the goal gradient hypothesis; Hull, 1932). Recent work has shown that people generally exert more cognitive effort when they are provided with information on their progress in a task (Devine & Otto, 2022; Katzir, Emanuel, & Liberman, 2020), and cognitive effort exertion increases near the end of a task (i.e., near goal completion, Devine et al., 2024; Emanuel, Katzir, & Liberman, 2022). Outside the laboratory context, the influence of perceived goal progress on motivation has also been documented. For instance, customers bought coffee more frequently the closer they were to getting a free coffee by collecting enough stamps in a reward program (Kivetz et al., 2006). In addition to influencing people’s motivation, goal progress can also influence the magnitude of the ‘frustration’ effect when they are blocked from the reward (Amsel, 1958). For instance, Haner and Brown (1955) found that children responded with more force when a failure occurred close to success, compared to when they were still far from achieving their goal.” *While facial EMG has indeed been used as an indicator of cognitive effort exertion, in our study we are mainly focussing on the fEMG after receiving the outcome rather than during the task. As such, the fEMG activities here primarily reflect affect triggered by outcomes, rather than cognitive effort exertion. We therefore did not expand on the relationship between fEMG and cognitive effort exertion in our manuscript.*  

*2. *One key element which I found unclear throughout the paper was *which *effects Eben and colleagues were aiming to replicate. In their Discussion they state: “Our RT data showed participants sped up in the arrow task the closer they got to the reward. Once they were blocked, participants seemed to be surprised about the outcome and confirmed the outcome with a slowed response [...O]ur current results are not in line with the original study” (p. 14). Yet, in reading Yu et al. (2014), they specifically de-emphasize the confirm-RTs in their analysis: “[Participants] were told that “Confirming the outcome is not a speeded task, and that you have plenty of time (4 sec) to make this response” (p. 167). Further, only RTs to arrows (not confirm-RTs) are reported in Yu et al.’s main text figures and tables. In fact, apart from listing average confirm-RTs, I am not sure where Yu et al. report confirm-RT results whatsoever. Thus, I am not entirely sure what to make of the discussed discrepancy in confirm-RTs. In any case, I am not sure it is correct to position the current replication as strongly inconsistent with the original findings—especially when the majority of behavioral and self-report measures do seem consistent. Perhaps the authors could further clarify their view on the importance of confirm-RTs to the original findings.

**Reply**: *As indicated on p.4 (highlighted in the revised version, see also below), we are mainly interested in outcome-related effects of frustration, which included the confirm RT, self-reported surprise, frustration and the confirm force (which due to technical issues was invalid, and hence not included in the manuscript). For consistency with the original work, we also examined the other variables, namely arrow RTs and self-reported motivation in the task.* “The ubiquitous influence of goal progress on motivated behavior requires a better understanding of how such influence arises. Here we specifically focus on how goal progress may influence the magnitude of the ’frustration’ effect when individuals are blocked from a reward.” Yu and colleagues indeed found no effects on confirm RTs. We think the confirm RT is still an important variable to consider though, for several reasons. First, much previous work on feedback-related behavioral adaptation uses response times as the main indicator, such as post-loss speeding and post-reinforcement pause in the gambling context (and in reinforcement learning more generally), and post-error slowing in the cognitive control field. Using confirm RTs allows us to potentially link the current finding to this body of work on response times. Second, specifically within the gambling context, how quickly people react to previous outcomes (which are captured by confirm RTs here) are related to event frequency, which is how many gambling events one may experience within a certain amount of time. Event frequency is an important structure feature of gambling products and has been proposed to play a key role in gambling-related harms. Examining the ‘frustration’ effect via confirm RTs therefore also has potential practical implications. *We agree with the reviewer that most of our results are consistent with the previous findings. For instance, when using the original analysis approach by Yu and colleagues, we also observed non-significant effects of proximity and expended effort on confirm RTs. For a better comparison between the current and the original results, we included the original plots in Figure 2. This figure shows that most of the behavioral results are highly consistent between the original and our replication study. It also highlights the discrepancy, namely the effects on surprise. The effect of proximity on surprise together with our exploratory analyses on confirm RTs suggest that there might be an orienting response to blocked rewards when the goal proximity was high. This is a finding that is not in line with the original findings. We adjusted our conclusion to weaken the claim that our findings are not in line with the original study.*  

*3.*Regarding statistical power, I think there is a bit more work to be done here. Firstly, I was not clear how the authors computed power for their analyses. They report a benchmark Cohen’s d (0.5) which they are able to detect with 80% power. But both their replication analyses (ANOVAs) and exploratory analyses (Bayesian MLMs) do not have effect sizes on this metrics. So perhaps a conversion was done?

**Reply**: We have adjusted our sample size justification to be completely transparent (see p. 6) *:* "In the original analysis, Yu and colleagues examined the effects of proximity and expended effort by combining the p values from multiple repeated-measures ANOVAs using the Stouffer’s method. As far as we know, no analytical method exists to compute the statistical power for the Stouffer’s combined p values. We therefore decided to go beyond the sample sizes used in Yu et al. (between 20 and 27), but still within our resource constraints. We were additionally interested in potential differences in the response force after wins and losses, for which Yu et al. did not report the results. We therefore assumed a medium effect size (Cohen’s d = 0.5), for which 32 participants would provide approximately 80% power to detect in a within-subject design. Our response force data, however, was compromised and we never followed up on that specific effect. The eventual sample size of 32 was therefore chosen given all these considerations, to (1) exceed the original sample sizes, (2) be within our resource constraints, and (3) provide sufficient power to detect potential response force differences between wins and losses.”  

 4.Leaving this question aside, the authors’ rightfully highlight that using Yu et al.’s ANOVA-based analysis, they too fail to find a significant confirm-RT effect (replicating the original null finding). However, when using a multilevel approach, this proximity effect on confirm-RTs emerges. I wholly agree that the multilevel approach is the superior one to the ANOVA-based analysis. However, calculating power in an MLM context is quite different than doing so for ANOVA and usually requires simulation. If indeed the MLM approach afforded increased power over the inferior ANOVA approach, then it could be that the original Yu et al. study was simply underpowered to detect the proximity effect on confirm-RTs. Again, it is hard to judge since, from what I can see, Yu et al. do not report or visualize any confirm-RT data in their original publication. But if this is indeed just a power issue (e.g., Yu et al also found numerical increases in confirm-RTs as a function of proximity, but which failed to achieve significance under an ANOVA approach), this would be a helpful piece of information for explaining the discrepancy in results.

**Reply**: *We discuss the possibility of a statistical power issue on p. 14.* *“*Note that when we used the same data analysis approach as in the original study, we also did not observe statistically significant effects of proximity or effort. This discrepancy in results might arise because the exploratory analysis reported here included proximity and effort simultaneously in one model and analyzed data on each trial (rather than aggregated means in each condition), and thus might have more statistical power to detect an effect.  Yu et al. did not report or visualize the results on confirm RTs, so it is unclear whether a similar pattern was also present in their data.”  

 5. Going beyond replication-by-significance, it would be interesting to see a brief discussion of relative effect sizes. It is difficult to compare right now because arrow-RT effects are reported by ANOVA in Yu et al. but by MLM in Eben et al. Nevertheless, eyeballing Figure 2 in Eben et al. against Figure 3 in Yu et al., the RT and self-report effects seem remarkably similar (e.g., a roughly ~40ms difference from 1/4 to 4/4 schedules in both samples). At the most, something like a small-telescopes approach could be used (Simonsohn, 2015), but even just a qualitative comparison of the relative effect sizes—particularly for the confirm-RTs (see power issue from comment #3 above)—could be very informative.

**Reply**: *We added the original study’s plots to our plots for a better comparison (p. 24). This plot now shows that for arrow RT, self-reported motivation, and self-reported frustration, the effect sizes were highly similar between the current study and Yu et al. It also highlights the difference in self-reported surprise. For confirm RT, unfortunately, since Yu et al. did not report detailed results, such a comparison is not possible.  We also added the analyses on the RTs to the arrows (as done in the original study) in our results table and in the main text (p.11). *  

 6. Some more details about the fEMG analysis would be helpful. What preprocessing steps were taken? Were signals bandpassed filtered, and if so at what frequency range? Were they rectified and low-pass filtered? (see van Boxtel, 2010).  Some of these cleaning steps may help more subtle effects emerge, especially under conditions of potentially low power as the authors highlight on p. 13.

**Reply**: *We report these specifics in the paragraph “Electrophysiological recording and data reduction” on p. 9.* “According to common guidelines and practices (Fridlund & Cacioppo, 1986; Korb et al., 2017), we attached shielded EMG electrodes over the right zygomaticus major and corrugator supercilii areas and placed a ground electrode at the top of the central forehead. Using the Biopac EMG100C amplifier with the MP150 module and the Acqknowledge software (www.biopac.com), we acquired continuous EMG at 1000 Hz sampling rate, with a 100 Hz high-pass and a 500 Hz low-pass. The data reduction and preprocessing pipeline was based on Korb et al. (2017), van Boxtel (2010) and Whelan (2003), and adjusted for our design. We applied a 20-200 Hz band pass filter and full-wave rectified the data by flipping all negative values of the signal to positive values. To exclude high-frequency noise, we then applied a 40 Hz low-pass filter. We epoched the data time-locked to the outcome presentation (win or block) from -750 ms to 2000 ms. Before cleaning the data, we compared the behavioral and EMG data to identify extra "ghost" triggers (triggers that Biopac picked up but were never sent) and deleted these. Then, we excluded error trials and trials where the baseline was more than 2 standard deviations from the participant’s baseline mean. The signal was normalized by expressing it as a percentage of the baseline mean per trial. This was done to ensure comparability between individuals and muscles, as well as to control for changes in muscle tone over time. Finally, we excluded the epochs with responses two standard deviations higher than the participant’s condition mean.”

 7. Additionally, here the authors adopted a grand-mean ERP approach for computing fEMG signals (p. 8). Instead, the authors could consider a single-trial approach, in which a summary value for each muscle is computed per trial per subject (e.g., the percent change in maximal constriction from baseline). This approach yields a single scalar summary value per trial and could mitigate power issues by keeping more data in the analysis and permitting a multilevel analysis similar to their RT analysis (something like: “cor ~ mo(proximity) + mo(effort) + ( mo(proximity) + mo(effort) | id)”). The downside to this approach is that it precludes a timecourse analysis, but as far as I understand, the timecourse of the EMG signal is less of interest than the comparison of experimental conditions in this case.  See Devine et al. (2023) for an example of this approach.  

**Reply**: *Following the reviewer’s suggestion, we took a single-trial approach. For each participant, muscle and trial, we found the maximum percent change from baseline in the 250 – 2000 ms window after outcome presentation.* *The trial-level corrugator activity data after blocked outcomes were then standardized within participants and analyzed with a mixed-effects model in brms. The pseudo-code for the brms model was: corrugator ~ mo(proximity) + mo(effort) + (mo(proximity) + mo(effort)|participant). We used the student’s t distribution as the likelihood function, to reduce the potential influence of extreme values. The coefficients for proximity and effort were not statistically credible, estimate = 0.002, 95% CI = [-0.038, 0.043], and estimate = -0.016, 95% CI = [-0.059, 0.022], respectively.* *We similarly analyzed the trial-level zygomaticus data after blocked outcomes. Here, the effect of effort was again not statistically credible, estimate = 0.004, 95% CI = [-0.023, 0.039]. The effect of proximity was statistically credible, estimate = 0.022, 95% CI = [0.002, 0.041], indicating that as participants were blocked proximal to a reward, the activity of zygomaticus tended to increase.* *As noted in the main text, the number of schedules within each condition was small, which was the reason why we decided to compare the win versus blocked outcomes, rather than examining the effects of different blocked schedules on fEMG data, as suggested here. Figures 5 and 7 also show large variations across participants in the fEMG data. It is thus unclear how valid and reliable the results obtained above are. For these reasons, we prefer to keep our original grand-mean ERP approach in the manuscript.*  

**Minor comments ---------------------**

  

1. Interestingly, the blocking procedure used here closely mirrors that used in classical experiments on goal-gradients in rats (Brown, 1948). I am not sure if this was an inspiration or not for the original design, but if not, it is an interesting coincidence.

**Reply**: *That is indeed very interesting. Since Yu and colleagues did not mention Brown (1948) at all, we assume that this is an interesting coincidence.*

  

1. I very much liked the video of the experiment uploaded to OSF! More researchers should do this—it makes evaluating the methodology much easier.

**Reply**: *Thanks so much for this feedback.*

  

1. Though Yu et al also did not analyze accuracy rates, I wonder if proximity or effort meaningfully influenced accuracy rates in this task. Or was accuracy too high and invariable for a simple task as this?

**Reply**: *As suggested, we analyzed accuracy as a function of proximity and effort, in a multilevel logistic regression. The pseudo-code for the *brms *model was: accuracy ~ mo(proximity) + mo(effort) + (mo(proximity) + mo(effort)|participant). The mo() function indicates that the variables were modelled as monotonic predictors. We observed that as proximity increased, accuracy decreased, estimate = -0.298, 95% CI = [-0.441, -0.160]. The effect of effort was not statistically credible, estimate = -0.068, 95% CI = [-0.201, 0.068]. These results, combined with the observation that participants tended to respond more quickly as proximity increased, suggest that proximity modulates the speed-accuracy trade-off. As they were getting close to a reward, they emphasized speed over accuracy, making responses more quickly at the cost of being less accurate.* *This result seems to differ from that of Devine et al. (2024), where participants responded more quickly, but not less accurately, when they were close to a reward. Our task put a strong emphasis on speed (i.e., beating the computer by responding quicker than an unknown deadline), and the arrow task was rather simple, potentially explaining the difference in the results. Devine et al. showed that their results were best explained by reduced response caution and increased information processing when participants were proximal to a goal. We speculate that in our task, proximity might similarly reduce response caution (thereby explaining the speed-accuracy trade-off), but did not strongly increase information processing due to the simple nature of the task. While this is not the focus of Yu et al. nor our current replication attempt, the task with the schedules used here might be a useful tool to disentangle the effects of proximity and expended effort in cognitive effort exertion.*
